# Long-term outcomes of untreated micropenis: growth patterns and predictive factors

**DOI:** 10.3389/fped.2025.1501259

**Published:** 2025-04-30

**Authors:** Davoud Amirkashani, Mostafa Abdollahi Sarvi, Mostafa Masoumi

**Affiliations:** ^1^ Division of Endocrinology and Metabolism, Ali Asghar Children Hospital, Iran University of Medical Sciences, Tehran, Iran; ^2^ School of Medicine, Iran University of Medical Sciences, Tehran, Iran; ^3^ School of Cognitive Sciences, Institute for Research in Fundamental Sciences (IPM), Tehran, Iran

**Keywords:** micropenis, penile length, endourology, penile growth, untreated micropenis

## Abstract

**Background:**

Micropenis, defined as a penile length more than 2.5 standard deviations below the mean for age and population, presents significant concerns for patients and parents. Despite current guidelines recommending multidisciplinary management, there is limited evidence on long-term outcomes, particularly in untreated patients.

**Methods:**

This prospective cohort study involved 46 male children aged 7–9 years presenting with micropenis at the Ali Asghar Endocrine Clinic from 2015 to 2023. Initial penile size, BMI, and other growth parameters were measured, with biannual follow-ups extending 3 years post-bone fusion to evaluate growth rates and influential factors.

**Results:**

Initial mean stretched penile length (SPL) was 3.22 ± 0.21 cm. Significant increases in penile size were observed across all intervals, with the highest growth rates occurring between the first- and second-years post-fusion. BMI emerged as the most significant predictor of penile growth, while initial SPL was the least influential factor. By the third-year post-fusion, the vast majority of subjects (44) achieved penile lengths within the normal range. Two patients, while showing penile growth over time, remained below the cutoff value defined by reference standards.

**Conclusion:**

Our findings indicate that most untreated micropenis patients reach normal penile size by adulthood, highlighting the importance of monitoring growth rates rather than focusing only on initial penile size. This study provides critical insights for developing guidelines and management strategies for micropenis, emphasizing the necessity of continued follow-up to ensure optimal outcomes.

## 1 Introduction

Penile length is a significant aspect of male anatomical and physiological health (such as sexual development, impacting not only sexual function but also psychological well-being and social dynamics or even regional cultures across time (1–3).

Previous studies have shown that factors such as BMI (body mass index), age of puberty onset, and the timing of bone fusion can significantly influence penile development and length (4, 5). These findings highlight the multifaceted nature of factors affecting penile growth and development. Additionally, penile length can serve as an indicator of various malformations, including hormonal imbalances, congenital anomalies, metabolic conditions, and anatomical defects (6).

With all that, there is an uprising of concern among parents and patients about penile length (1, 7). As a result, urologists and endocrinologists are facing an increasing number of patients with concerns about short penis. The majority of these individuals have penile lengths that fall within the normal range according to established guidelines and do not require medical intervention (1). However, a subset of these patients lies in the micropenis rage and do present with genuine concerns that show the importance of further evaluation and potential treatment.

Micropenis is defined as a penile length more than 2.5 standard deviations below the mean for age and population (8). Current guidelines recommend a multidisciplinary approach involving endocrinologists, urologists, and psychologists, with treatments often such as hormonal therapy (6, 8, 9). However, there is a lack of robust evidence supporting the long-term efficacy and safety of these treatments and also the outcome of patients who didn't receive the treatment. Additionally, existing guidelines do not provide clear criteria for when or how physicians should initiate or withhold treatment, underscoring the need for comprehensive guidelines that include long-term follow-up to provide valuable and consistent care for patients with micropenis, particularly those who have not undergone treatment (1).

As we recognize the gap in studies among patients not receiving treatment, our aim is to longitudinally follow up these patients from their initial assessment through post-puberty, examining the impact of somatometric factors. Additionally, we aim to demonstrate that patients who do not receive treatment often achieve normal penile size by the conclusion of the study period.

## 2 Materials and methods

### 2.1 Study design

We initially enrolled 82 male children, aged 7–9 years, presenting with micropenis as the chief complaint brought by their parent. This prospective cohort study was conducted at the Ali Asghar Endocrine Clinic from 2015 to 2023, with follow-up visits occurring twice a year. However, due to the long-term follow-up design, the number of participants decreased over time. The final number of participants included in the analysis process was 46, reflecting the attrition rate throughout the study period.

None of the boys included had congenital anomalies, metabolic diseases or any other background visits (1). All boys who met the criterion of micropenis by having a stretched penile length shorter than the 2.5 SD less than the mean length, according to “the 2011 age-matched New York Cohen children medical center of north shore- long island” (10), penile length standards, were included in the study (2, 11, 12).

All measurements were conducted by the same observer (first author, NT). The prepubic subcutaneous fat at the base of the penis was compressed with one end of the ruler down to the pubic ramus. The observer then fully stretched the penis by holding the glans between the left thumb and index finger, while the ruler was placed along the side of the stretched penis using the observer's right hand. The length from the lower edge of the pubic bone to the tip of the glans (excluding foreskin) was measured. It's important to mention that all of the subjects were circumcised.

Based on the 6 months visit schedule, in the first visit, we documented the weight, height and calculated BMI and age at the first visit. During subsequent visits, testicular size and volume were assessed by the same observer (first author). The onset of puberty was determined when testicular size reached 2.5 cm or a volume of 4 cc, as measured using an orchidometer.

Additionally, the age of bone fusion was determined by examining the growth charts (Iran National Growth Chart provided by the Ministry of Health of Iran) and using wrist x-ray imaging when the growth rate approached zero or plateaued. Patients were followed up for 3 years after identifying the age of bone fusion, with assessments conducted at 1-year intervals, during which penile size was measured.

### 2.2 Ethical considerations

At the beginning of the study, the research protocol was thoroughly explained to the parents, and informed consent was obtained. During each visit or examination, one or both parents were present to observe the examination.

The protocol for this study was reviewed and approved by the Institutional Review Board of Iran University of Medical Sciences (approval number: 05-2024-155).

### 2.3 Statistical analysis

Quantitative variables are expressed as mean ± standard deviation, while categorical variables are presented as frequency (percentage). Statistical analyses were conducted using the *t*-test and linear mixture model with the Python statistical libraries NumPy 2.0.1 (13) and SciPy1.140. A *p*-value less than 0.05 was considered statistically significant.

## 3 Results

### 3.1 Characteristics of patients

The characteristics of the patients presenting with concerns regarding micropenis are detailed in Table 1. A total of 46 patients, with an age range of 7–9 years (mean ± SD, 8.04 ± 0.77 years), were included in the study. The initial stretched penile length (SPL) for all patients was measured at 3.22 ± 0.21 cm (Table 1).

**Table 1:** Patient characteristics: this table summarizes the demographic and clinical characteristics of the patients, including first visit age, weight, and body mass index (BMI), and penile sizes [from first visit (FV) to 3-year post-fusion (3y/f)] with developmental stage ages.

Characteristics	Min	Max	Median	Mean	Standard deviation (std)
First visit age (year)	7	9	8	8.04	0.77
Height (cm)	113	135	124.5	125.02	6.07
Weight (kg)	18	34	24.5	25.34	3.92
BMI	12.71	20.43	16.19	16.15	1.67
Size at first visits	2.8	3.8	3.2	3.22	0.21
Puberty age	9	12	11	10.67	0.88
Puberty size	4.2	6.4	5.4	5.45	0.44
Fusion age	14.9	17	15.9	15.82	0.57
Fusion size	6.9	14	11.05	11.14	1.33
1/y post fusion	7.5	14.6	11.6	11.76	1.24
2/y post fusion	7.5	15	13.65	13.31	1.37
3/y post fusion	7.9	15	14	13.91	1.32

### 3.2 Penile size changes over time

**Figure 1:**
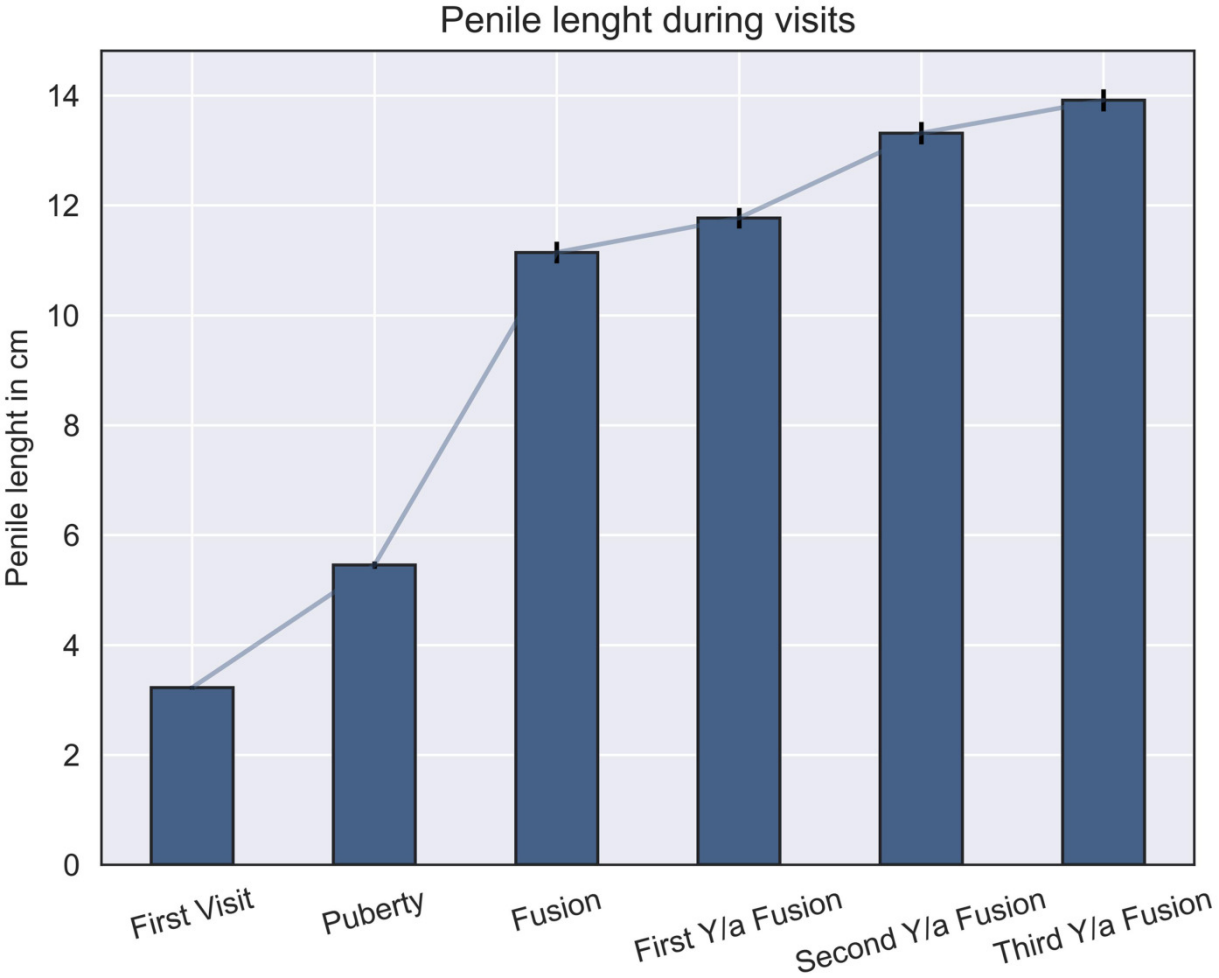
Comparison of penile length across different visits and developmental stages: this figure illustrates the penile lengths at various developmental checkpoints, showing significant differences between all stages (*p* ≤ 0.05).

As illustrated in Figure 1 and Table 2, there is a significant increase in penile size observed from the initial visit to the third-year post-fusion. Additionally, significant increments are evident in penile length from the first visit to puberty, continuing from puberty to fusion, and further progressing from fusion to the final follow-up visit (Figure 1, Table 2).

**Table 2:** Mean penile length and standard error at different checkpoints: the table presents the mean penile length (in cm) and corresponding standard error across various stages, including the first visit, puberty, fusion, and subsequent yearly follow-ups after fusion.

Checkpoints	Mean penile length (cm)	Standard error
First visit	3.22	0.03
Puberty	5.45	0.06
Fusion	11.14	0.19
First year/a fusion	11.76	0.18
Second year/a fusion	13.31	0.2
Third year/a fusion	13.91	0.19

### 3.3 Growth rates

**Figure 2:**
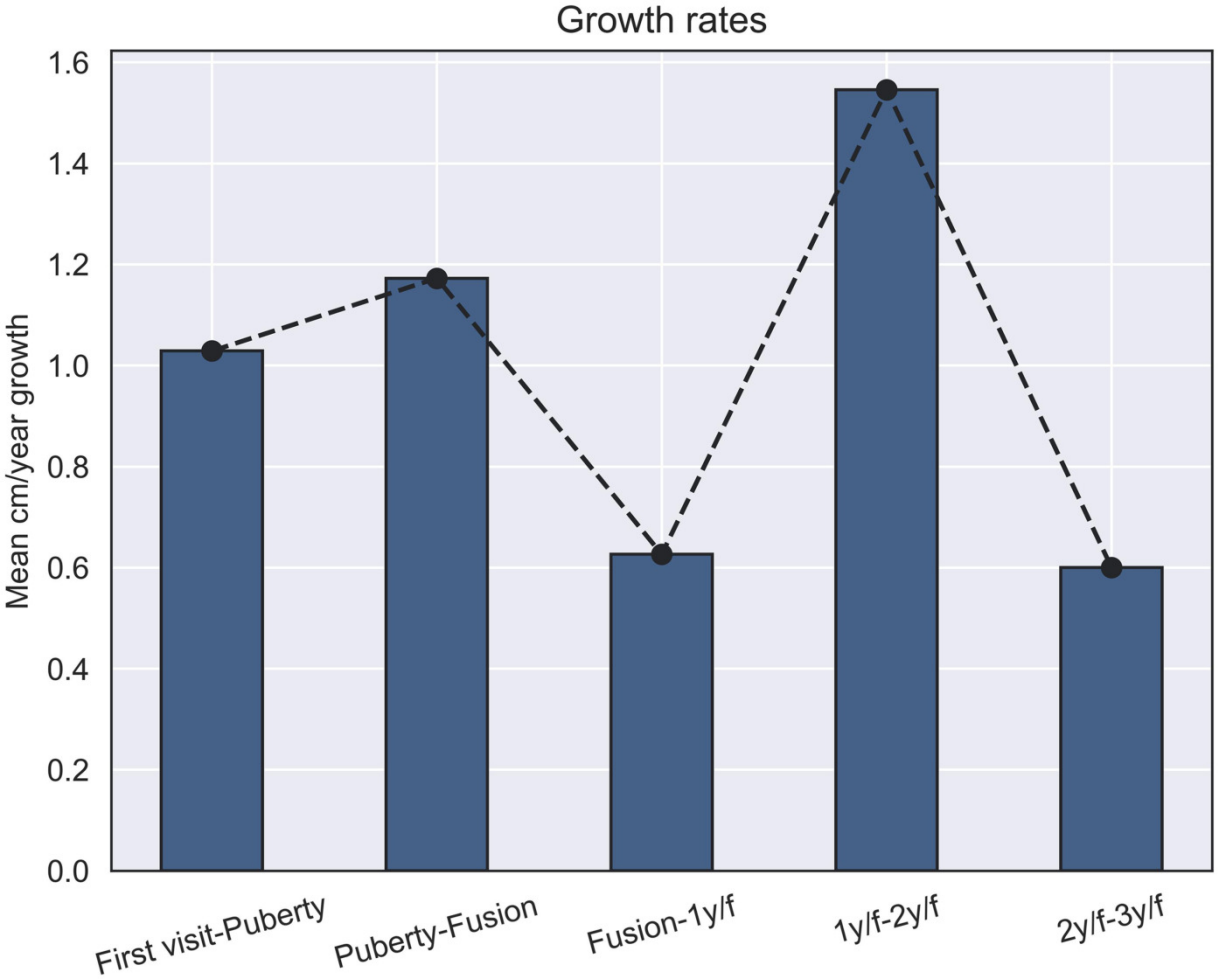
Penis growth rates (cm) across developmental stages: this figure shows the growth rates per year of penile length in centimeters within different developmental stages, with significant differences observed between all stages (*p* ≤ 0.05).

As depicted in Figure 2 and Table 3, it is evident that all growth rates are statistically significant and differ significantly from each other. The most rapid increase in penile size occurs between the first and second years following fusion (1), while the smallest increment is observed between the second- and third-years post-fusion (We dropped the size of penis as first, second and third year after fusion; Figure 2, Table 3).

**Table 3:** Annual growth rates of penile length between checkpoints: this table shows the computed growth rates of penile length (in cm per year) between different developmental stages.

Checkpoints	Growth rate per year (cm)
First visit-puberty	1.02
Puberty-fusion	1.17
Fusion-1y/f	0.62
1y/f–2y/f	1.54
2y/f–3y/f	0.6

### 3.4 Effective factors

**Figure 3:**
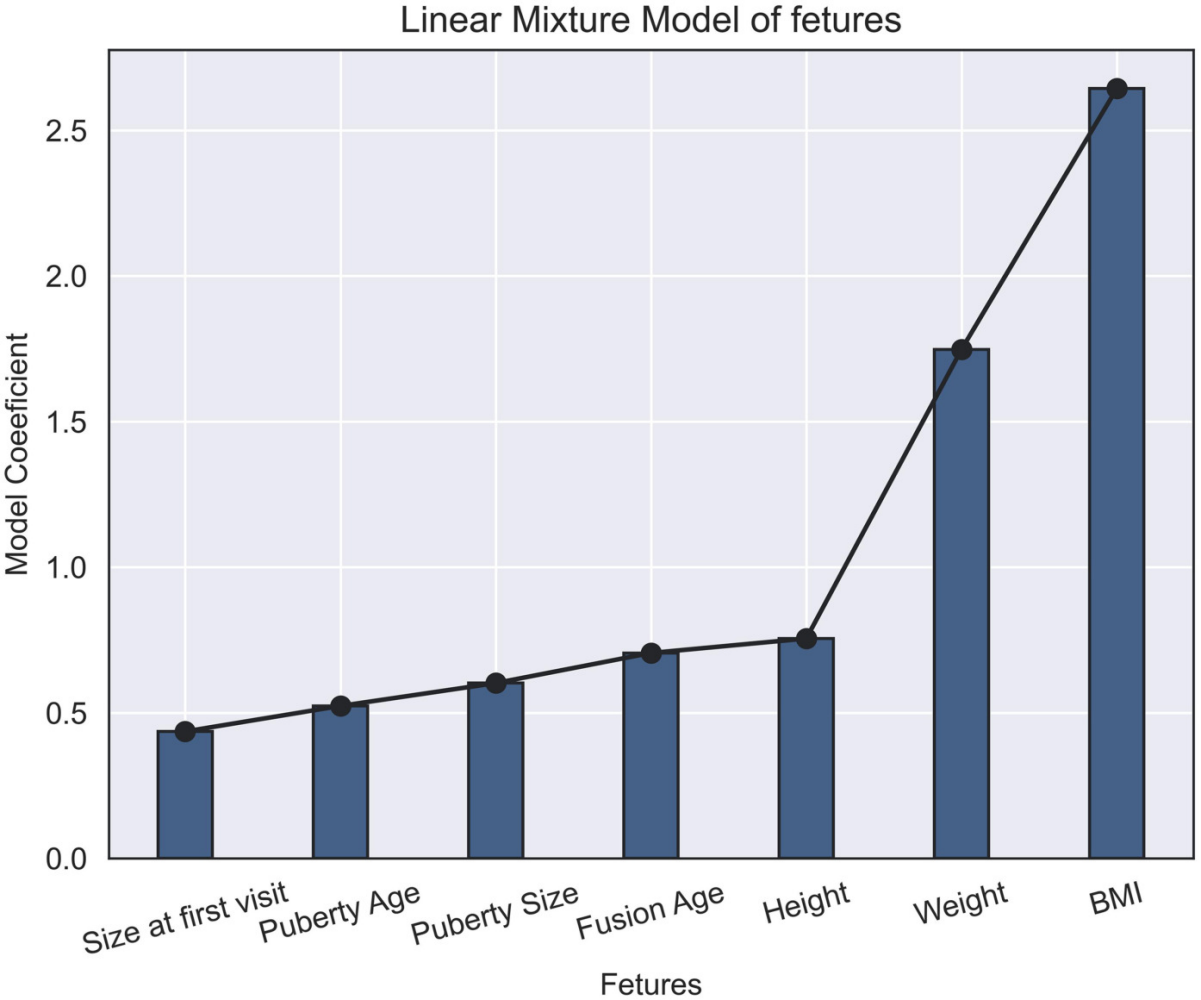
Linear mixture model of penile growth predictors: the model shows BMI as the most significant predictor of penile growth, while initial penile size at the first visit had the least influence.

The linear mixture model facilitated the extraction of the most influential factors, with BMI emerging as the most significant predictor. Conversely, the initial penile size at the first visit was identified as the least influential factor in determining subsequent growth trajectories (Figure 3).

**Figure 4:**
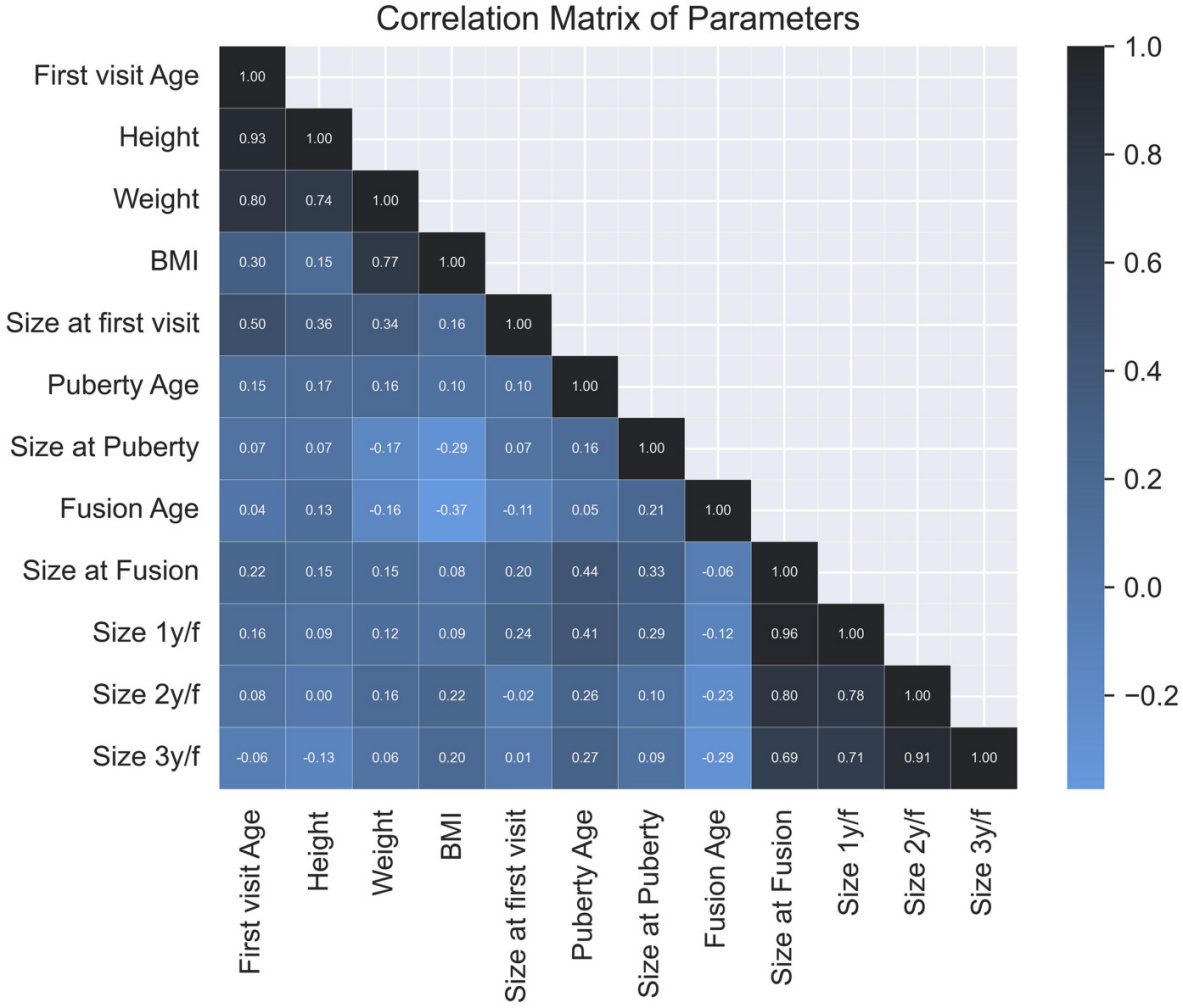
Correlation matrix of study parameters: this heatmap visualizes the relationships between key variables, such as BMI, puberty onset, bone fusion, and penile length measurements profiles.

The correlation matrix elucidates the relationships between various parameters used in this study (Figure 4). This heatmap allows for the visualization and analysis of correlations among key variables, including BMI, age of puberty onset, hormonal profiles, and penile length measurements. The matrix provides valuable insights into the interdependencies and potential associations among these factors within the context of penile growth and development.

## 4 Discussion

The aim of our study was to conduct a long-term follow-up of patients with micropenis who did not receive any treatment and to demonstrate that most of these patients will achieve normal penile size as they grow, including 3 years post-bone fusion. Our findings indicated that the majority of our sample from our population (44 subjects) will reach normal penile size by puberty [with the metrics of the New York penile length chart (10)]. Furthermore, penile length continues to increase after puberty, extending into the post-bone fusion period (1). By the third-year post-fusion, our comparisons reveal that the vast Majority of our cohort surpasses the minimal normal range of penile length based on Iranian penile length standards too (3). Although most participants reached penile lengths within the normal range by the third-year post-fusion, two individuals, despite demonstrating steady penile growth across all timepoints, did not fully meet the cutoff criteria for normal SPL as defined by the New York reference chart (10). This variability emphasizes the need for personalized long-term monitoring, even in the presence of positive growth trends.

One of the most crucial factors in properly following up with patients is monitoring the growth rate of penile size during the intervals between visits. Our study identified that the highest growth rates occur between the first- and second-years post-fusion. The growth rates for other intervals, in descending order, are: puberty to fusion, the first visit to puberty, fusion to the first-year post-fusion, and lastly, from the second-year post-fusion to the third-year post-fusion. Although two subjects reached a normal penile size by puberty, their growth rates between puberty and bone fusion were insufficient compared to the population, underscoring the necessity of continued follow-up even after achieving normal penile size. This finding indicates to physicians that only a few patients with true micropenis will require treatment, emphasizing the importance of ongoing monitoring to ensure proper development Additionally, physicians can use these growth rate intervals as checkpoints for patient follow-up.

As previously mentioned, there are correlation factors that assist physicians in predicting patient penile size growth and prognosis. These factors are crucial for determining the optimal timing for treatment initiation to achieve the best outcomes with minimal side effects during both childhood and adulthood. BMI emerged as the most significant predictor of penile growth. The correlation analysis showed a significant relationship between BMI and penile growth (*p* < 0.01). The linear regression model confirmed BMI as the most influential factor, with a standardized regression coefficient of 2.5 (Figure 3). In contrast, initial penile size at the first visit was the least influential factor in determining subsequent growth trajectories according to the linear mixture model, suggesting not losing hope in treatment even with small measurements at the first visits.

There are several limitations in our study that should be mentioned. The first limitation is the study population. Due to the requirement for long-term follow-up and multiple visits, the number of participants decreased over the years (1). Secondly, there were exogenous factors beyond our control, such as nutritional and economic conditions, although no proven nutritional deficiencies were identified (1). Lastly, the study was conducted over 7 years, preventing us from continuing follow-up for the fourth- and fifth-years post bone fusion.

Future studies should aim to conduct this research on a larger population (1) and consider a longer follow-up period, particularly extending into adulthood, to analyses long-term physical and psychological effects. Additionally, we recommend that future researchers establish comparative studies between our data and those of individuals receiving hormonal therapy.

This study offers valuable insights into penile growth among the micropenis population from the prepubertal to post-puberty stages. The growth rates and the correlation coefficients previously discussed provide a guideline and checkpoints for physicians, aiding in optimal patient management. The most significant finding of our study is that the growth rate of penile size, rather than the penile size itself, serves as the primary parameter of interest. This focus on growth rate is crucial for guiding clinical decisions and ensuring effective patient care.

## Data Availability

The raw data supporting the conclusions of this article will be made available by the authors, without undue reservation.
